# High‐intensity mindfulness and cognitive behavioral group therapy for social anxiety disorder: Preliminary efficacy

**DOI:** 10.1002/pcn5.70264

**Published:** 2025-12-07

**Authors:** Shota Noda, Kentaro Shirotsuki, Yoshio Kodama, Mutsuhiro Nakao, Hisanobu Kaiya

**Affiliations:** ^1^ Department of Psychology Philipps University of Marburg Marburg Germany; ^2^ Research Institute of Cognitive Behavior Therapy Musashino University Koutou‐Ku Tokyo Japan; ^3^ Faculty of Human Sciences Musashino University Koutou‐ku Tokyo Japan; ^4^ Tokorozawa Mental Clinic Tokorozawa‐shi Saitama Japan; ^5^ Institute of Stress Management Showa Medical University Shinagawa‐ku Tokyo Japan; ^6^ Panic Disorder Research Center Warakukai Medical Corporation Minato‐ku Tokyo Japan

**Keywords:** cognitive behavioral therapy, high‐intensity program, mindfulness, social anxiety disorder

## Abstract

**Aim:**

Cost and probability biases are known to predict improvements in symptoms of social anxiety disorder (SAD). This study developed a high‐intensity mindfulness and cognitive behavioral group therapy (M‐CBT) program—comprising mindfulness training, psychoeducation, cognitive restructuring, exposure exercises, and group　sharing—designed to reduce these biases. The intervention was delivered in a group format, and its preliminary efficacy in patients with SAD was evaluated in a pilot single‐arm trial.

**Methods:**

Patients (*N* = 10) diagnosed with SAD through a structured clinical interview participated in an eight‐session M‐CBT program. They completed a set of questionnaires assessing SAD symptoms, cost and probability biases, fear of negative evaluation, self‐focused attention, depressive symptoms, subjective happiness, dispositional mindfulness, cognitive reappraisal, and suppression at pre‐intervention, mid‐intervention, post‐intervention, and follow‐up.

**Results:**

Analyses using linear mixed‐effects models revealed that high‐intensity M‐CBT produced improvements in SAD symptoms, cost and probability biases, depressive symptoms, subjective happiness, dispositional mindfulness, and cognitive reappraisal (*p* < 0.05). The intervention also yielded significant reductions in cost and probability biases from pre‐ to post‐treatment and at follow‐up, with large effect sizes (cost bias: Cohen's *d* = 0.85–1.27; probability bias: *d* = 1.07–2.42). In contrast, the effect sizes for SAD symptoms were moderate (*d* = 0.57–0.67).

**Conclusions:**

These findings suggest that high‐intensity M‐CBT can alleviate SAD symptoms and reduce cost and probability biases. Moreover, delivering M‐CBT in a high‐intensity format appears to be effective for individuals with SAD. Future randomized‐controlled trials are warranted to more rigorously confirm these effects.

## INTRODUCTION

Social anxiety disorder (SAD) is characterized by intense anxiety in social situations, a persistent fear of negative evaluation (FNE) by others, and avoidance of social interactions.^1^ SAD has a relatively high prevalence^2^ and is frequently comorbid with major depressive disorder (MDD). In addition to this comorbidity, it is also linked to marked difficulties in social, academic, and occupational domains, together with a pronounced decline in overall quality of life.^3^, ^4^ Cognitive behavioral therapy (CBT) is widely regarded as the gold‐standard psychological treatment for SAD. Meta‐analytic findings reported by Mayo‐Wilson et al.^5^ indicate a large effect size for individual CBT (SMD = 1.19) and a somewhat smaller, though still substantial, effect for group CBT (SMD = 0.92), both of which fall within the large effect range. However, despite its efficacy, a substantial number of individuals fail to achieve clinically meaningful improvement following CBT. For example, a meta‐analysis by Springer et al.^6^ examining remission rates of SAD among adults (aged 18 years and older) treated with CBT found that only 40.1%–40.4% achieved remission. Therefore, there is significant room for improvement in the treatment.

Improvements in cost and probability biases have been shown to predict reductions in SAD symptoms. Moscovitch et al.^7^ examined changes in clinical outcomes across three time points (pre‐, mid‐, and post‐treatment) in individuals with SAD, comparing those who responded to CBT with those who did not. Their findings revealed notable differences in changes related to cost and probability biases. Specifically, treatment responders showed significant reductions in both types of cognitive bias, whereas non‐responders did not show such improvements. Cost bias refers to the tendency to overestimate the severity or consequences of negative social events.^8^ In contrast, probability bias denotes the exaggerated estimation of their likelihood.^8^ Cost and probability biases have been shown to play a significant role in the maintenance of social anxiety.^9^ Foa et al.^8^ reported that reductions in cost bias were strong predictors of improvement in SAD symptoms. In contrast, Calamaras et al.^10^ found that decreases in probability bias were more strongly associated with symptom reduction. Given these mixed findings regarding the most relevant cognitive predictors of treatment response in SAD, recent research has applied network analysis to better clarify these relationships. Noda et al.^11^ suggest that cost bias may be more strongly associated with reductions in SAD symptoms in non‐clinical populations, such as university students, whereas probability bias may be a more salient predictor of SAD symptoms in individuals with a clinical diagnosis of SAD.

In recent years, third‐wave CBT‐related interventions incorporating mindfulness concepts and mindfulness training (MT) have been reported to be effective in the treatment of SAD and are considered potentially beneficial even for patients who have not responded to traditional CBT.^12^ Mindfulness‐based interventions (MBIs), as well as CBT approaches that include mindfulness components, have demonstrated efficacy in improving social anxiety, FNE, self‐focused attention (SFA), cost and probability biases, and avoidance behaviors.^13^, ^14^, ^15^, ^16^, ^17^ Noda et al.^18^ and Schmertz et al.^19^ found that improvements in dispositional mindfulness were positively associated with reductions in cost bias, probability bias, and social anxiety, suggesting the effectiveness of MT in reducing cost and probability biases. However, Noda et al.^11^ emphasized the importance of addressing these symptoms not only through MT but also by directly targeting them using cognitive behavioral techniques, as probability bias and social anxiety were identified as central symptoms in individuals with SAD.

Noda et al.^16^, ^20^ developed a low‐intensity mindfulness and cognitive behavioral group therapy (M‐CBT) by integrating MT with cognitive restructuring, with the aim of targeting cost and probability biases and alleviating symptoms of SAD. In a randomized‐controlled trial (RCT), Noda et al.^20^ evaluated the intervention's efficacy in individuals with a high level of SAD symptoms. The results demonstrated that, compared to the control group, participants who received low‐intensity M‐CBT showed significant improvements in cost and probability biases related to negative cognitions when attending to others, FNE, depressive symptoms, dispositional mindfulness, and subjective happiness. Additionally, Noda et al.^16^ found that low‐intensity M‐CBT produced significant reductions in cost and probability biases, FNE, SFA, and symptoms of SAD and MDD among patients diagnosed with SAD. However, low‐intensity M‐CBT is positioned as an adjunctive treatment module and is expected to serve as an effective additional option for patients with SAD who require further intervention targeting cost and/or probability biases, as well as for individuals with other mental disorders who experience high social anxiety symptoms and may benefit from targeted support. To date, no high‐intensity version of M‐CBT—designed to reduce cost and probability biases and alleviate symptoms of SAD—has been established as a standalone treatment, nor has any such intervention been empirically demonstrated to yield large and durable treatment effects.

Therefore, the present study aimed to develop a high‐intensity M‐CBT protocol and to examine its preliminary efficacy in patients with SAD. This high‐intensity protocol expanded upon the low‐intensity M‐CBT framework by incorporating additional exposure therapy components. Alex Brake et al.^21^ suggested that mindfulness may be a prerequisite for optimizing the outcomes of exposure therapy. Enhancing mindfulness fosters greater awareness and acceptance of internal experiences and strengthens individuals' capacity to confront anxiety‐provoking situations.^22^ Specifically, mindful emotion awareness, or nonjudgmental and present‐focused attention toward emotions, may facilitate engagement in exposures, which may, in turn, enhance therapeutic outcomes such as reductions in anxiety symptoms and avoidance.^23^ To date, several studies have supported the effectiveness of combining MT with exposure therapy for individuals with SAD.^15^, ^24^, ^25^ For instance, England et al.^24^ found that mindfulness‐ and acceptance‐based exposure was more effective in achieving diagnostic remission than exposure guided by a habituation rationale. Such programs typically include MT and techniques aimed at cultivating acceptance and cognitive defusion, which are practiced both before and during exposure exercises. Based on this evidence, we hypothesized that combining MT, cognitive restructuring, and exposure therapy would result in a highly effective intervention for reducing cost and probability biases, as well as alleviating clinical symptoms of SAD. To test this hypothesis, we conducted a pilot single‐arm trial in which the high‐intensity M‐CBT protocol was administered to individuals diagnosed with SAD.

## METHODS

### Participants and procedure

**Figure 1:**
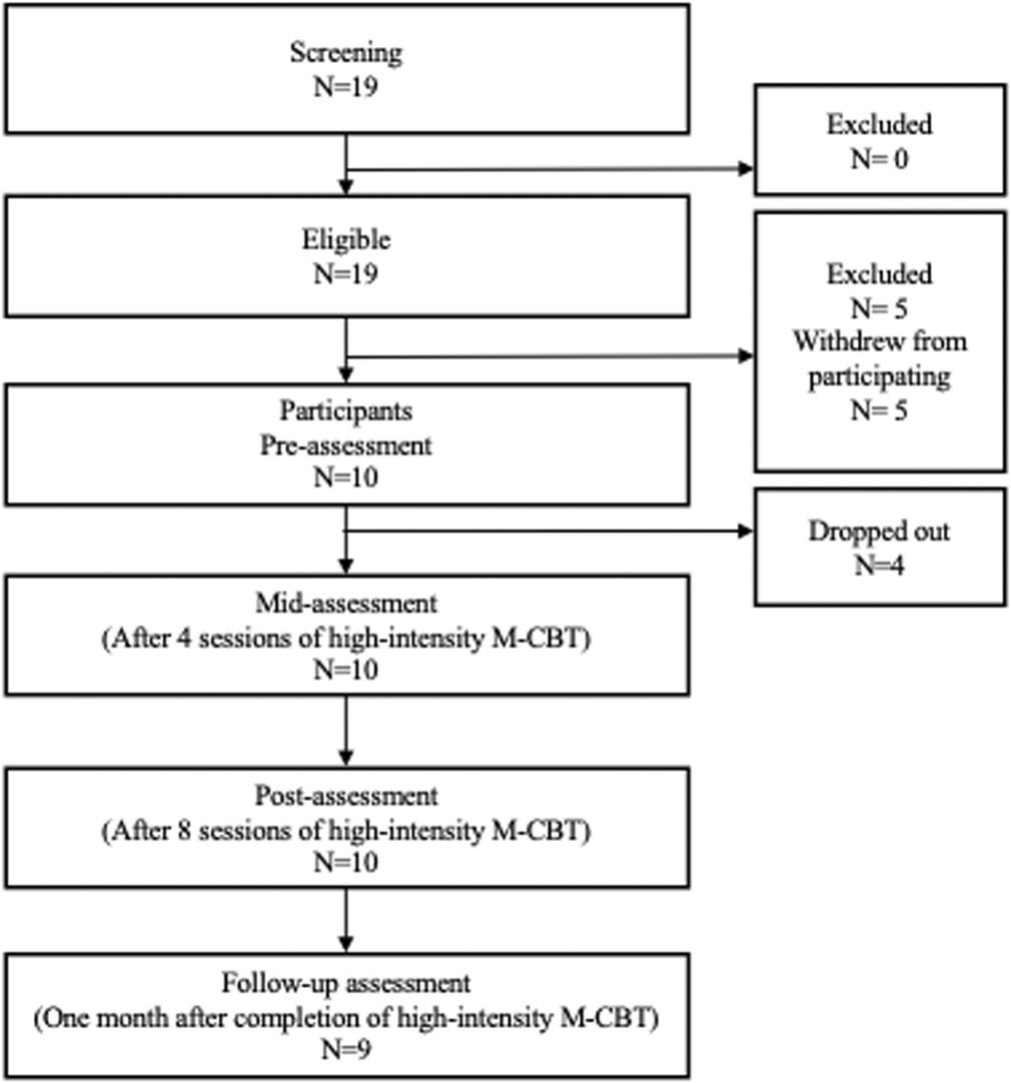
Participants' flowchart. *Note*: The program was delivered in a group format over eight weekly sessions. M‐CBT, mindfulness and cognitive behavioral group therapy.

The study recruited adult outpatients, primarily presenting with symptoms of SAD, from two psychiatric outpatient clinics in Japan. Participants were eligible if they received a diagnosis of SAD based on DSM‐5 criteria,^26^ confirmed through the Mini‐International Neuropsychiatric Interview 7.0.2 (MINI).^27^ Participants also had to be 18 years of age or older. Individuals were excluded if they had current psychotic disorders or bipolar disorders, were experiencing a manic episode, demonstrated a high risk of suicide, suffered from a severe physical illness, or had significant cognitive impairments. To ensure safety, each patient's health status was evaluated by their attending psychiatrist, and only those who were referred and approved by the psychiatrist were invited to participate. A total of 19 individuals expressed interest in participating and were subsequently screened through individual interviews to determine their eligibility. The first author, certified as both a licensed public psychologist and a clinical psychologist, conducted the diagnostic assessments using the MINI in face‐to‐face sessions. All those who met the diagnostic and inclusion criteria provided written informed consent prior to enrollment in the study. Of the initial 19 participants, five discontinued their participation before beginning high‐intensity M‐CBT due to either COVID‐19–related concerns or scheduling difficulties. Among the remaining participants, 10 (Mage = 42.70, SD = 10.57; 3 men, 7 women) completed the intervention, while four dropped out during the course of treatment due to scheduling difficulties, diminished interest in study participation, or changes in treatment. Participants were asked to self‐report any negative physical or mental changes before each session, and each session was conducted only after confirming their psychological stability. A detailed flow of participant progress is presented in Figure 1. Of the 10 patients, 2 had a current major depressive episode, and 5 had had a past major depressive episode. Five patients had previously received CBT, and six were undergoing pharmacological treatment for their clinical symptoms. Medications included alprazolam (*n* = 1), aripiprazole (*n* = 1), atomoxetine hydrochloride (*n* = 1), brotizolam (*n* = 1), clonazepam (*n* = 1), clotiazepam (*n* = 1), etizolam (*n* = 2), flutoprazepam (*n* = 1), fluvoxamine maleate (*n* = 1), lorazepam (*n* = 1), lurasidone hydrochloride (*n* = 1), rilmazafone hydrochloride hydrate (*n* = 1), sertraline hydrochloride (*n* = 1), and zolpidem tartrate (*n* = 1). The average duration of treatment at the clinics was 1107.00 days (±1705.48), which reflects the inclusion of chronic patients who had been receiving treatment for more than a few years.

**Figure 2:**
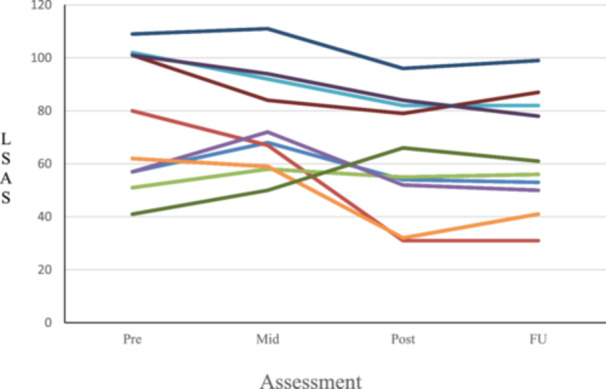
Changes in Liebowitz Social Anxiety Scale (LSAS) scores across pre‐, mid‐, post‐treatment, and follow‐up assessments.

This study was approved by the Research Ethics Committee of the Faculty of Human Sciences, Musashino University (Approval Number: 20192403), and was registered in the UMIN Clinical Trial Registration System (UMIN: 000039834).

### Measures

#### Clinician‐administered structured interview

Mental disorders were evaluated using the MINI 7.0.2,^27^ in line with DSM‐5 diagnostic standards. In this study, assessments focused on SAD, major depressive episodes, bipolar disorders, and psychotic disorders. The structured interview was administered based on screening indicators both prior to study inclusion and following the intervention.

#### Self‐reported questionnaire outcomes

The following measures were assessed as treatment outcomes at four time points: pre‐intervention, mid‐intervention (after four sessions), post‐intervention, and 1‐month follow‐up.

#### Primary self‐reported outcomes

##### The Japanese version of the Liebowitz Social Anxiety Scale

The Japanese version of the Liebowitz Social Anxiety Scale (LSAS)^28^ was administered to evaluate the severity of SAD symptoms. This self‐report questionnaire assesses fear and avoidance behaviors in socially anxiety‐inducing situations. The LSAS contains 24 fear items and 24 avoidance items, each scored on a four‐point Likert scale ranging from 0 to 3. A total score (0–144) is calculated, with subscale scores of up to 72 for both fear and avoidance, where higher values indicate greater symptom severity.

##### The Speech Cost/Probability bias Scale

The Speech Cost/Probability bias Scale (SCPS)^29^ was administered to measure cognitive biases related to speech situations, specifically cost and probability biases. The SCPS comprises 11 items assessing cost bias and 11 items assessing probability bias, each rated on a five‐point Likert scale ranging from 1 to 5. Total scores range from 11 to 55, with higher values representing stronger cost and probability biases.

#### Secondary self‐reported outcomes

##### The Short Form of the Japanese Fear of Negative Evaluation Scale

FNE, a core diagnostic feature of SAD, was measured using the Short Form of the Japanese Fear of Negative Evaluation Scale (SFNE).^30^ The SFNE comprises 12 items rated on a five‐point Likert scale (1–5), yielding total scores ranging from 12 to 60. Higher scores indicate greater apprehension about being judged negatively by others.

##### The Japanese version of the Self‐Focused Attention Scale

SFA, considered a central maintaining mechanism of SAD, was assessed using the Japanese version of the Self‐Focused Attention Scale (SFAS).^31^ The SFAS consists of 11 items comprising two subscales: six items assessing the arousal factor and five items assessing the behavioral factor. Each item is rated on a five‐point scale (0–4), producing a total score ranging from 0 to 44, with higher scores indicating a stronger tendency toward SFA.

##### The Japanese version of the Self‐rating Depression Scale

The Japanese version of the Self‐rating Depression Scale (SDS)^32^ was administered to evaluate MDD symptom severity. The SDS is composed of 20 items, each answered on a four‐point response format (1–4). Possible scores range from 20 to 80, with higher values denoting more pronounced MDD symptoms.

##### The Japanese version of the Subjective Happiness Scale

The Japanese version of the Subjective Happiness Scale (SHS)^33^ is a self‐report measure of subjective happiness. The scale consists of four items, each rated on a seven‐point scale. Item 4 is reverse‐scored. Total scores range from 4 to 28, with higher scores reflecting greater levels of subjective happiness.

##### The Japanese version of Five Facet Mindfulness Questionnaire

The Japanese version of the Five Facet Mindfulness Questionnaire (FFMQ)^34^ was used to assess dispositional mindfulness. The questionnaire includes 39 items distributed across five subscales: Observing (8 items), Acting with Awareness (8 items), Nonjudging (7 items), Nonreactivity (8 items), and Describing (8 items). An overall score, obtained by summing the five subscales, served as an index of dispositional mindfulness. Each item is rated on a five‐point Likert scale (1–5), yielding total scores from 39 to 195, with higher scores reflecting greater mindfulness.

##### The Japanese version of Emotion Regulation Questionnaire

The Japanese version of the Emotion Regulation Questionnaire (ERQ)^35^ was used to measure emotion regulation strategies, focusing on cognitive reappraisal and suppression. The cognitive reappraisal subscale contains six items, while the suppression subscale contains four items, each rated on a seven‐point Likert scale from 1 to 7. Total scores range from 6 to 42 for cognitive reappraisal and from 4 to 28 for suppression, with higher scores indicating greater reliance on each respective strategy.

#### Therapy protocol

High‐intensity M‐CBT is an enhanced program that supplements the four sessions of low‐intensity M‐CBT with an additional four mindfulness‐based exposure sessions. The mindfulness‐based exposure conducted in this study is not traditional exposure therapy based on the habituation rationale or the expectancy violation approach; rather, it aims to foster mindful awareness and acceptance of one's own internal reactions in the exposed social situation. The program was delivered in a group format over eight weekly sessions, each lasting 90–120 min, across an 8‐week period. The program primarily consisted of MT, psychoeducation, cognitive restructuring, exposure exercises, and group sharing. The therapeutic protocol is detailed in Table 1. Sessions 1–4 followed the same procedures as in previous studies.^16^, ^20^ The purpose of Session 5 was to increase awareness of participants' social anxiety in social situations. The session consisted of MT (mindful yoga and/or sitting meditation), identification of avoidance patterns, mindful exposure, and group sharing. First, participants practiced MT to become aware of their internal responses prior to exposure therapy. They then received an explanation of exposure therapy and its effects, followed by the identification of four types of avoidance patterns in social situations: cognitive, emotional, physical, and behavioral. Subsequently, each participant engaged in an individual speech exposure exercise. MT was conducted both before and after exposure to help participants observe their internal reactions. Finally, participants shared their experiences with the group. The purpose of Session 6 was to directly experience social anxiety in social situations. The session included MT, creation of a record sheet to track internal reactions (thoughts, emotions, physical sensations, and behaviors), mindful exposure, and group sharing. As in Session 5, MT, mindful exposure, and group sharing were implemented. However, before the exposure exercise, participants completed a mental reaction record sheet, and after the exposure exercise, they reviewed the sheet to identify constructive thoughts. The purpose of Session 7 was to practice acceptance of participants' reactions in social situations. The session consisted of MT (mindful yoga and/or sitting meditation), creation of a record sheet to track internal reactions, mindful interoceptive exposure, and group sharing. Through yoga practice, participants increased their awareness of interoceptive sensations and were instructed to maintain attention to these sensations during speech exposure. The purpose of Session 8 was to enhance participants' ability to be themselves in social situations. The session consisted of MT (mindful yoga and/or sitting meditation), program reflection, loving‐kindness meditation, and group sharing. Participants reflected on the overall program and shared what had changed, what had not changed, and what they hoped to change in the future. Each session included meditation (sitting, imagery, or loving‐kindness meditation) as homework, and participants were instructed to practice for at least 20 min daily. To facilitate adherence, audio recordings of approximately 20‐min guided meditations were provided.

**Table 1:** Overview of a high‐intensity mindfulness and cognitive behavioral group therapy (M‐CBT) protocol.

Session	Title	Intervention techniques	Homework
1	Discover the factors that are increasing social anxiety	Mindful yoga	Sitting meditation
		Developing a treatment plan	Informal mindfulness practice
		Psychoeducation	Diary of daily happiness
		Sitting meditation	
		Group sharing	
2	Identify the factors that are causing social anxiety	Mindful yoga	Sitting meditation or imagery meditation
		Psychoeducation	Informal mindfulness practice
		Imagery meditation	Diary of thoughts, emotions, behaviors, and physical reactions when interacting with others
		Group sharing	
3	Observe the factors that are causing social anxiety	Developing an anxiety hierarchy list and personal version of cognitive behavioral models	Sitting meditation or imagery meditation
		Imagery meditation	Informal mindfulness practice
		Group sharing	Diary of communication with others
4	Let go of the factors that are causing social anxiety	Imagery meditation	Sitting meditation, imagery meditation, or loving‐kindness meditation
		Cognitive restructuring	Informal mindfulness practice
		Loving‐kindness meditation	
		Group sharing	
5	Becoming aware of your social anxiety in social situations	Mindful yoga or sitting meditation	Sitting meditation
		Identifying your avoidance patterns	Informal mindfulness practice
		Mindful exposure	Mindfully experiencing everyday social situations
		Group sharing	
6	Experiencing your social anxiety in social situations	Mindful yoga or sitting meditation	Sitting meditation or imagery meditation
		Creating a record sheet of your internal reactions (thoughts, emotions, physical sensations, and behaviors)	Informal mindfulness practice
		Mindful exposure	Mindful exposure diary
		Group sharing	
7	Accepting your reactions in social situations	Mindful yoga or sitting meditation	Sitting meditation or imagery meditation
		Creating a record sheet of your internal reactions (thoughts, emotions, physical sensations, and behaviors)	Informal mindfulness practice
		Mindful interoceptive exposure	Mindful exposure diary
		Group sharing	
8	Being yourself in social situations	Mindful yoga or sitting meditation	Sitting meditation, imagery meditation, or loving‐kindness meditation
		Program reflection	Informal mindfulness practice
		Loving‐kindness meditation	
		Group sharing	

All group sessions were facilitated by the first author holding certifications both as a licensed public psychologist and as a clinical psychologist. The program was conducted in three cohorts, with three, two, and five participants completing each cohort, respectively.

### Statistical analyses

The effectiveness of high‐intensity M‐CBT was evaluated using linear mixed‐effects models. For each outcome measure, when a significant main effect of time was observed, post hoc comparisons were conducted with Bonferroni correction. As this was a pilot study, all analyses were considered exploratory, and Bonferroni corrections were applied within each outcome rather than across all outcomes. Effect sizes for changes from pre‐treatment to post‐treatment or follow‐up were quantified using Cohen's *d*. All statistical analyses were carried out in spss version 28.0 (IBM Corp., Armonk, NY, USA).

Regarding missing data, one participant had two missing values on the LSAS at the follow‐up assessment. The corresponding item score from the post‐test was substituted based on the last observation carried forward (LOCF) method. Additionally, one participant did not complete the follow‐up assessment; for this case, the entire post‐test score set was carried forward as the follow‐up score using the LOCF approach.

## RESULTS

### Treatment effects

Table 2 reports the descriptive statistics (means and standard deviations) of each outcome variable together with the results from the linear mixed‐effects models. Analyses indicated significant decreases across all primary outcomes (LSAS: *F*(3, 27) = 4.84, *p* < 0.01, Figure 2; LSAS‐Fear: *F*(3, 27) = 4.87, *p* < 0.01; LSAS‐Avoidance: *F*(3, 27) = 3.35, *p* < 0.05; SCPS–Cost bias total score: *F*(3, 27) = 11.82, *p* < 0.01; SCPS–Cost bias in the negative cognition from one's own performance: *F*(3, 27) = 8.15, *p* < 0.01; SCPS–Cost bias in the negative cognition that occurs by paying attention to others: *F*(3, 27) = 10.57, *p* < 0.01; SCPS–Probability bias total score: *F*(3, 27) = 12.90, *p* < 0.01; SCPS–Probability bias in the negative cognition from one's own performance: *F*(3, 27) = 8.15, *p* < 0.01; SCPS–Probability bias in the negative cognition that occurs by paying attention to others: *F*(3, 27) = 9.90, *p* < 0.01). Significant reductions were also observed in several secondary outcomes (SFNE reverse‐scored item factor: *F*(3, 27) = 3.43, *p* < 0.05; SFAS total score: *F*(3, 27) = 3.51, *p* < 0.05; SFAS‐Arousal: *F*(3, 27) = 3.23, *p* < 0.05; SDS: *F*(3, 27) = 10.05, *p* < 0.01; SHS: *F*(3, 27) = 6.07, *p* < 0.01; FFMQ: *F*(3, 27) = 13.05, *p* < 0.01; ERQ–Cognitive reappraisal: *F*(3, 27) = 13.05, *p* < 0.01). In contrast, no significant improvements were found in other secondary outcomes (SFNE: *F*(3, 27) = 2.74, *p* = 0.06; SFNE straightforwardly worded item factor: *F*(3, 27) = 2.07, *p* = 0.13; SFAS‐Behavior: *F*(3, 27) = 2.88, *p* = 0.05; ERQ–Suppression: *F*(3, 27) = 0.63, *p* = 0.60).

**Table 2:** Means and standard deviations of outcomes and results of linear mixed‐effects models.

		1	2	3	4	*F*‐values	Multiple comparisons
		Pre‐test	Mid‐test	Post‐test	Follow‐up test		
LSAS total score	Mean (SD)	76.10 (25.38)	75.50 (19.20)	63.10 (22.13)	63.80 (21.81)	4.84**	3, 4 < 1*
Fear	Mean (SD)	42.90 (11.05)	44.20 (10.06)	37.50 (11.94)	37.10 (11.80)	4.87**	
Avoidance behavior	Mean (SD)	33.20 (15.05)	31.30 (10.03)	25.60 (10.66)	26.70 (10.75)	3.35*	3 < 1*
SCPS cost bias total score	Mean (SD)	40.60 (8.75)	40.40 (6.72)	33.40 (10.22)	32.40 (8.96)	11.82**	3, 4 < 1**
Negative cognition from one's performance	Mean (SD)	27.80 (7.58)	27.80 (5.18)	23.10 (7.64)	22.50 (6.74)	8.15**	3, 4 < 1**
Negative cognition generated when paying attention to others	Mean (SD)	12.80 (2.20)	12.60 (2.07)	10.30 (2.70)	9.90 (2.51)	10.57**	3, 4 < 1**
SCPS probability bias total score	Mean (SD)	37.50 (8.44)	31.50 (4.25)	26.60 (7.85)	25.40 (7.12)	12.90**	3, 4 < 1** 2 < 1*
Negative cognition from one's performance	Mean (SD)	25.80 (7.44)	21.50 (3.57)	18.80 (5.43)	17.30 (5.10)	8.15**	3, 4 < 1**
Negative cognition generated when paying attention to others	Mean (SD)	11.70 (2.79)	10.00 (1.70)	7.80 (2.53)	8.10 (2.33)	9.90**	3, 4 < 1**
SFNE total score	Mean (SD)	52.40 (6.29)	52.20 (5.22)	44.70 (12.09)	47.40 (9.76)	2.74	
Straightforwardly worded item factor	Mean (SD)	34.80 (4.47)	34.80 (3.26)	29.40 (8.20)	32.20 (8.09)	2.07	
Reverse‐scored item factor	Mean (SD)	17.60 (2.22)	17.40 (2.55)	15.30 (4.22)	15.20 (3.36)	3.43*	
SFAS total score	Mean (SD)	32.10 (5.61)	32.10 (6.94)	27.70 (6.18)	28.20 (8.03)	3.51*	
Arousal	Mean (SD)	15.40 (4.97)	16.60 (6.19)	13.40 (4.09)	13.50 (5.48)	3.23*	
Behavior	Mean (SD)	16.70 (2.58)	15.50 (1.84)	14.30 (3.53)	14.70 (3.53)	2.88	
SDS	Mean (SD)	56.90 (5.02)	53.50 (6.96)	48.60 (6.79)	47.80 (7.39)	10.05**	3, 4 < 1**
SHS	Mean (SD)	11.40 (2.84)	14.40 (2.72)	15.70 (3.77)	15.60 (2.68)	6.07**	3, 4 < 1** 2 < 1*
FFMQ	Mean (SD)	98.10 (21.46)	104.80 (19.79)	120.20 (20.60)	123.00 (21.92)	13.05**	3, 4 < 1**
ERQ cognitive reappraisal	Mean (SD)	22.50 (7.58)	27.90 (4.70)	27.20 (7.69)	27.60 (5.44)	5.34**	2, 4 < 1** 3 < 1*
ERQ suppression	Mean (SD)	16.80 (3.39)	16.80 (3.36)	15.90 (3.35)	16.00 (3.16)	0.63	

Abbreviations: ERQ, Emotion Regulation Questionnaire; FFMQ, Five Facet Mindfulness Questionnaire; LSAS, Liebowitz Social Anxiety Scale; SCPS, Speech Cost/Probability bias Scale; SDS, Self‐rating Depression Scale; SFAS, Self‐Focused Attention Scale; SFNE, Short Fear of Negative Evaluation Scale; SHS, Subjective Happiness Scale.

* *p* < 0.05.

** *p* < 0.01.

### Effect sizes

Table 3 summarizes the effect sizes comparing the pre‐test with the post‐test and follow‐up assessments. From pre‐ to post‐test, significant effect sizes were found for SCPS–Cost bias, SCPS–Probability bias, SDS, SHS, FFMQ, and ERQ–Cognitive reappraisal (*p* < 0.05). In the comparison between pre‐test and follow‐up, significant effect sizes were observed for SCPS–Cost bias, SCPS–Probability bias, SFNE (reverse‐scored item factor), SDS, SHS, FFMQ, and ERQ–Cognitive reappraisal (*p* < 0.05).

**Table 3:** Within‐group effects sizes for each outcome.

	Pre‐mid effect sizes (Cohen's *d*)	95% CI	Pre–post effect sizes (Cohen's *d*)	95% CI	Pre‐follow‐up effect sizes (Cohen's *d*)	95% CI
LSAS total score	0.05	−0.57 to 0.67	0.65	−0.05 to 1.32	0.67	−0.04 to 1.35
Fear	−0.27	−0.90 to 0.37	0.57	−0.11 to 1.23	61	−0.09 to 1.27
Avoidance behavior	0.22	−0.42 to 0.84	0.60	−0.09 to 1.27	0.59	−0.10 to 1.25
SCPS cost bias total score	0.04	−0.59 to 0.66	1.04*	0.24–1.80	1.23*	0.37–2.04
Negative cognition from one's performance	0.00	−0.62 to 0.62	0.85*	0.10–1.56	0.99*	0.20–1.73
Negative cognition generated when paying attention to others	0.14	−0.49 to 0.76	0.98*	0.20–1.73	1.27*	0.41–2.10
SCPS probability bias total score	0.69	−0.02 to 1.37	1.38*	0.48–2.25	2.42*	1.14–3.67
Negative cognition from one's performance	0.51	−0.17 to 1.16	1.07*	0.27–1.85	1.91*	0.83–2.96
Negative cognition generated when paying attention to others	0.55	−0.13 to 1.21	1.30*	0.43–2.14	1.25*	0.39–2.08
SFNE	0.04	−0.58 to 0.66	0.66	−0.04 to 1.33	0.58	−0.11 to 1.24
Straightforwardly worded item factor	0.00	−0.62 to 0.62	0.67	−0.04 to 1.34	0.35	−0.30 to 0.99
Reverse‐scored item factor	0.12	−0.50 to 0.74	0.61	−0.09 to 1.27	0.73*	0.01–1.42
SFAS total score	0.00	−0.62 to 0.62	0.69	−0.02 to 1.37	0.61	−0.09 to 1.27
Arousal	−0.25	−0.87 to 0.39	0.54	−0.14 to 1.19	0.47	−0.19 to 1.12
Behavior	0.69	−0.02 to 1.36	0.67	−0.04 to 1.34	0.63	−0.07 to 1.30
SDS	0.67	−0.04 to 1.35	1.34*	0.45–2.18	1.43*	0.52–2.31
SHS	−1.09*	−1.87 to −0.28	−0.99*	−1.74 to −0.21	−0.95*	−1.68 to −0.17
FFMQ	−0.60	−1.26 to 0.10	−1.12*	−1.90 to −0.30	−1.20*	−2.01 to −0.36
ERQ cognitive reappraisal	−1.21*	−2.02 to −0.37	−0.87*	−1.59 to −0.12	−0.79*	−1.49 to −0.06
ERQ suppression	0.00	−0.62 to 0.62	0.43	−0.23 to 1.07	0.23	−0.41 to 0.85

* *p* < 0.05.

Abbreviations: CI, confidence interval; ERQ, Emotion Regulation Questionnaire; FFMQ, Five Facet Mindfulness Questionnaire; LSAS, Liebowitz Social Anxiety Scale; SCPS, Speech Cost/Probability bias Scale; SDS, Self‐rating Depression Scale; SFAS, Self‐Focused Attention scale; SFNE, Short Fear of Negative Evaluation Scale; SHS, Subjective Happiness Scale.

#### MINI‐assessed diagnostic changes in SAD and comorbid depression

Following high‐intensity M‐CBT, nine out of ten participants no longer met the diagnostic criteria for SAD as assessed by the MINI. Additionally, two participants were diagnosed with comorbid major depressive episodes prior to treatment; however, post‐intervention, neither met the diagnostic criteria for major depressive episodes.

#### Self‐reported negative physical or mental changes before each session

Only 1 out of 10 participants reported that the speech exposure was psychologically burdensome. The first author provided individual psychological care (supportive assistance) before, during, or after the session, after explaining again that the speech exposure was not mandatory but optional, and that the purpose was not to deliver a perfect speech but to observe one's own emotional reactions when speaking in front of others. That participant completed the program.

## DISCUSSION

The purpose of this study was to develop a high‐intensity version of M‐CBT and to examine its preliminary effectiveness for SAD using a pilot single‐arm trial. High‐intensity M‐CBT differs from second‐wave CBT in that it incorporates MT, which aims to help individuals notice and accept their internal reactions in social situations. The program was delivered in a group format and consisted of eight sessions, integrating Noda et al.'s^16^, ^20^ low‐intensity M‐CBT program with mindfulness‐based exposure therapy, which aims to foster mindful awareness and acceptance of one's own internal reactions during exposure to social situations. This enhanced program supplements the four sessions of low‐intensity M‐CBT with an additional four mindfulness‐based exposure sessions.

The primary self‐reported outcomes were SAD symptoms (measured by the LSAS) and cost and probability biases (measured by the SCPS). Linear mixed‐effects models revealed significant reductions in SAD symptoms, cost bias, and probability bias. Multiple comparisons further showed significant improvements in these outcomes from pre‐assessment to post‐assessment and follow‐up. High‐intensity M‐CBT led to significant pre–post and follow‐up improvements in cost and probability biases, with large effect sizes on the SCPS (cost bias: Cohen's *d* = 0.85–1.27; probability bias: *d* = 1.07–2.42). These results suggest that high‐intensity M‐CBT is effective in reducing cost and probability biases, in line with the program's intended targets. Notably, the intervention was particularly effective in addressing probability bias, which plays a central role in the maintenance and exacerbation of SAD symptoms.^11^ These effect sizes may be comparable to, or larger than, those reported by Shirotsuki et al.^36^ for a six‐session individual therapy program using traditional CBT techniques targeting cost and probability biases in Japanese patients with SAD (cost bias: *d* = 0.87; probability bias: *d* = 0.67), and by Hofmann^37^ for a 12‐session traditional group CBT in American patients with SAD (cost bias: *d* = 0.92). However, because of the limited sample size, definitive conclusions cannot be drawn from this study. Future research should use larger samples to more robustly evaluate the effectiveness of high‐intensity M‐CBT.

Although the effect sizes for SAD symptoms were moderate (*d* = 0.57–0.67), they did not reach statistical significance due to the small sample size. These effect sizes may be comparable to those reported by Chen et al.^38^ for a group CBT program using traditional CBT techniques based on the Clark and Wells model in Japanese patients with SAD, although the sample size in the present study was considerably smaller. In their study, the effect size for the LSAS total score in the intention‐to‐treat sample was 0.61, calculated as (M_pre – M_post)/SD_pre). The average number of sessions per group was 15 (range: 11–20), which was greater than that of the high‐intensity M‐CBT program. The effect sizes observed in the present study were also somewhat larger than those reported by Shirotsuki et al.^36^ for SAD symptoms (*d* = 0.32–0.55) in the six‐session program based on individual CBT, but smaller than the effect size reported by Yoshinaga et al.^39^ for a 14‐session individual CBT program based on the Clark and Wells model in Japanese patients with SAD (*d* = 1.71). The effectiveness of the individual CBT program based on the Clark and Wells model has also been confirmed in an RCT.^40^ Taken together, high‐intensity M‐CBT appears to be effective for patients with SAD who have elevated cost and probability biases. While its efficacy might be comparable to that of group CBT based on the Clark and Wells model, individual CBT might offer greater benefits in reducing overall SAD symptom severity. Nevertheless, given the differences in the timing, sample size, and study context between the present and previous research, future RCTs are warranted to rigorously evaluate the efficacy of high‐intensity M‐CBT and other CBT approaches.

The secondary self‐reported outcomes of this study were FNE, SFA, MDD symptoms, subjective happiness, dispositional mindfulness, and emotion regulation. Linear mixed‐effects models identified significant changes in the reverse‐scored factor of SFNE, SFA, MDD symptoms, subjective happiness, dispositional mindfulness, and cognitive reappraisal. Multiple comparisons indicated significant improvements in MDD symptoms, subjective happiness, dispositional mindfulness, and cognitive reappraisal between the pre‐intervention assessment and both the post‐intervention and follow‐up assessments. Ninomiya et al.^41^ conducted mindfulness‐based cognitive therapy (MBCT) with 20 Japanese patients diagnosed with either panic disorder or SAD, reporting an increase in total FFMQ scores from a baseline of 107.8 (SD = 14.3) to 119.2 (SD = 17.5), with an effect size of *d* = –0.71. In contrast, the present study showed relatively larger effect sizes for dispositional mindfulness between the pre‐test and both the post‐test and follow‐up (*d* = –1.20 to –1.12), although the limited sample size precludes definitive conclusions. These findings suggest that high‐intensity M‐CBT may be particularly effective in enhancing dispositional mindfulness in individuals with SAD. The effect sizes for symptoms of MDD were also notably large (*d* = 1.34–1.43). These effect sizes are comparatively greater than those reported for traditional individual CBT by Shirotsuki et al.^36^ for MDD symptoms (*d* = 0.71), as well as those reported for MBCT by Piet et al.^17^ among Danish patients with SAD (*d* = 0.64). Moreover, two participants who met the diagnostic criteria for a major depressive episode at baseline no longer met the criteria following the intervention. These findings suggest that high‐intensity M‐CBT may be effective in alleviating depressive symptoms comorbid with SAD. Subjective happiness and cognitive reappraisal demonstrated substantial effect sizes from the mid‐treatment point onward, in contrast to the other outcome measures. These changes may be partially attributable to the cognitive restructuring and loving‐kindness meditation introduced during the fourth session, which are presumed to have contributed to improvements in these outcomes.

Following high‐intensity M‐CBT, only one in ten participants met the diagnostic criteria for SAD according to the MINI (remaining diagnosis rate: 10%). However, as Springer et al.^6^ noted, while remission is often defined as no longer meeting diagnostic criteria for the disorder under treatment, this definition has an important limitation: patients may fall below the threshold simply because one symptom is absent, and yet still experience substantial and debilitating symptoms. An alternative approach therefore conceptualizes remission as scoring below a clinical cutoff on a continuous measure. Goldin et al.^14^ identified a reduction of 14 or more points in LSAS total scores as a reliable change. Such a reduction was observed in five participants after the intervention (reliable change response rate: 50.00%). In contrast, one participant's LSAS score increased by 25 points, although they no longer met the criteria for an SAD diagnosis according to the MINI. Notably, their dispositional mindfulness score increased by 37 points. This pattern suggests that the program may have contributed to the increase in LSAS scores by heightening the participant's awareness of anxiety related to social situations. Goldin et al.^14^ also defined scores below 48 as a clinically significant change, while Asakura et al.^28^ reported a cutoff of 44 points on the Japanese version of the LSAS for clinical SAD groups in Japan. Only two participants met both criteria—an LSAS reduction greater than 14 points and a post‐intervention score below both 44 and 48 (clinically significant change rate: 20.00%).

Taken together, high‐intensity M‐CBT appears to be an effective intervention for individuals with SAD, particularly those with elevated cost and probability biases, and may be especially beneficial for individuals with comorbid MDD. However, its effectiveness in achieving full remission of SAD symptoms may be limited.

There are several limitations to this study. First, the sample size was limited to only ten participants, which restricts the generalizability of the findings. To more robustly assess the efficacy of high‐intensity M‐CBT, future research with larger sample sizes is essential. Second, the study used a single‐arm design without a control group. Although the linear mixed‐effects model indicated significant improvements following the intervention, the absence of an RCT means that improvements could be partially attributed to factors such as the passage of time or placebo effects. Future studies should therefore include a control condition, such as a wait‐list group, to better isolate the treatment's effect. Third, six participants in this study were undergoing pharmacological treatment, and the study did not control for the potential effects of these medications. Future research should evaluate the efficacy of high‐intensity M‐CBT while controlling for the influence of pharmacological treatment. Fourth, the individuals responsible for implementing the program were identical to those who administered the MINI. Future research should examine outcomes when the program is implemented by personnel different from those conducting the diagnostic assessments, in order to minimize potential bias, such as the influence of the assessor's expectations on participants' performance or responses. Fifth, this study did not compare M‐CBT to standard CBT protocols that emphasize cognitive restructuring and exposure techniques. While we hypothesized that combining MT with conventional CBT techniques—such as cognitive restructuring and exposure exercises—might amplify their therapeutic effects, particularly in reducing cost and probability biases in SAD, this assumption remains untested in the absence of a direct comparison. Finally, some participants in this study withdrew before the program began due to COVID‐19–related concerns. Moreover, as the study was conducted after the COVID‐19 pandemic, appropriate infection‐control precautions were taken. These factors may have influenced both the feasibility and the generalizability of the findings. Future research conducted under more stable conditions will be essential to clarify the robustness and broader applicability of high‐intensity M‐CBT.

## CONCLUSION

This study introduced a high‐intensity version of M‐CBT and explored its preliminary efficacy in individuals diagnosed with SAD. The results indicated improvements across multiple domains, including SAD symptoms, cost and probability biases, MDD symptoms, subjective happiness, dispositional mindfulness, and cognitive reappraisal. These findings suggest that high‐intensity M‐CBT may serve as a practical treatment option for individuals with SAD, particularly those with pronounced cost and probability biases, and may be especially beneficial for those with comorbid MDD. To establish the effectiveness of this approach more robustly, future studies should use RCT designs.

## AUTHOR CONTRIBUTIONS

Shota Noda contributed to the study conception and design, performed the intervention program and assessment, material preparation, data collection, and analysis, and wrote the first draft of the manuscript. All authors commented on previous versions of the manuscript, and read and approved the final manuscript.

## CONFLICT OF INTEREST STATEMENT

The authors declare no conflicts of interest.

## ETHICS APPROVAL STATEMENT

The study protocol received ethical approval from the Research Ethics Committee of the Faculty of Human Sciences, Musashino University (approval number: 20192403).

## PATIENT CONSENT STATEMENT

All participants provided written informed consent before taking part in the study.

## CLINICAL TRIAL REGISTRATION

Trial registration number: University Hospital Medical Information Network (UMIN CTR) UMIN000039834. Registered May 16, 2020.

## CONSENT FOR PUBLICATION

Not applicable.

## Data Availability

The data that support the findings of this study are available from the corresponding author upon reasonable request.
